# Tweet Analysis for Enhancement of COVID-19 Epidemic Simulation: A Case Study in Japan

**DOI:** 10.3389/fpubh.2022.806813

**Published:** 2022-03-31

**Authors:** Vu Tran, Tomoko Matsui

**Affiliations:** ^1^Risk Analysis Research Center, The Institute of Statistical Mathematics, Tokyo, Japan; ^2^Department of Statistical Modeling, The Institute of Statistical Mathematics, Tokyo, Japan

**Keywords:** COVID-19, SEIR model, simulation, SNS, Twitter, emotion, emoji

## Abstract

The COVID-19 pandemic, which began in December 2019, progressed in a complicated manner and thus caused problems worldwide. Seeking clues to the reasons for the complicated progression is necessary but challenging in the fight against the pandemic. We sought clues by investigating the relationship between reactions on social media and the COVID-19 epidemic in Japan. Twitter was selected as the social media platform for study because it has a large user base in Japan and because it quickly propagates short topic-focused messages (“tweets”). Analysis using Japanese Twitter data suggested that reactions on social media and the progression of the COVID-19 epidemic may have a close relationship. Analysis of the data for the past waves of COVID-19 in Japan revealed that the relevant reactions on Twitter and COVID-19 progression are related repetitive phenomena. We propose using observations of the reaction trend represented by tweet counts and the trend of COVID-19 epidemic progression in Japan and a deep neural network model to capture the relationship between social reactions and COVID-19 progression and to predict the future trend of COVID-19 progression. This trend prediction would then be used to set up a susceptible-exposed-infected-recovered model for simulating potential future COVID-19 cases. Experiments to evaluate the potential of using tweets to support the prediction of how an epidemic will progress demonstrated the value of using epidemic-related social media data. Our findings provide insights into the relationship between user reactions on social media, particularly Twitter, and epidemic progression, which can be used to fight pandemics.

## 1. Introduction

We investigated the potential of using data from social media to enhance the prediction and simulation of an epidemic's progression. A case study was carried out using Twitter data related to the COVID-19 epidemic in Japan. The COVID-19 pandemic has been causing global problems that have affected everyone for a lengthy period, and the end is not in sight. During the pandemic, people tend to seek information or clues for use in deciding their next actions through a variety of channels: newspapers, TV, and especially social media (1, 2). Neely et al. (1) showed that in a questionnaire survey of 1003 US-based adults, 76% of the respondents relied on social media at least “a little,” and 59% of the respondents read information about COVID-19 on social media at least once per week, 63.6% of the respondents were unlikely to do fact-checking with a healthcare professional. Dadaczynski et al. (2) found that, in a cross-sectional study among university students in Germany, 37.6% (5,302/14,092) of the respondents use social media sometime or frequently for searching information on COVID-19 and related issues.

Studies have shown that, even long before the COVID-19 pandemic, social media greatly affects society, and could reflect social mental states (3–5). Work by Settanni et al. (3) analyzing Facebook posts revealed that, overall, the expression of negative emotions positively correlated with anxiety, depression, and stress symptoms and negative emotion usage positively correlated with anxiety symptoms. Park et al. (4) found that the use of words related to negative emotions and anger significantly increased among Twitter users with major depressive symptoms compared to those otherwise. Wald et al. (5) showed that it is possible to predict the factors in Big 5 Personality Index (6) (Agreeableness, Conscientiousness, Extroversion, Neuroticism, and Openness) and those in the Dark Triad (7) (Psychopathy, Machiavellianism, Narcissism) by using user posts on Twitter with rather good accuracy (AUC of 0.736).

Twitter is an attractive data source for analysis for several reasons: it is one of the largest social media platforms worldwide, it greatly affects several aspects of society (daily conversations, news reports, event advertisements, etc.) in various domains (health, entertainment, economics, research, politics, etc.), it makes user posts accessible by everyone, and it enables a tremendous amount of information to be easily accessed and shared. During the COVID-19 pandemic especially, a large volume of information on Twitter regarding the infection situation, symptoms, treatment, vaccinations, restrictions, and so on is being continuously shared and discussed. Users can share their emotions and opinions regarding the information instantaneously without geographical limitations. The effects of these emotions and opinions can thus spread rapidly. As shown in the collected data in a later section, the average number of daily tweets containing selected COVID-19 related keywords has been more than 400,000 during the COVID-19 epidemic in Japan.

Research on predicting the progression of the COVID-19 pandemic has received much attention worldwide (8). Early prediction is important for implementing countermeasures against its spread. Epidemiological models, e.g., the susceptible-exposed-infected-recovered (SEIR) model, are commonly used for such prediction. The parameters are obtained from observed data or set on the basis of predefined scenarios. Complex problems, e.g., the emergence of new variants, diverging government policies (9, 10), and diverging public perceptions (11, 12), have arisen as the pandemic has lasted longer and longer. Many countries, including Japan, have already experienced more than four waves of the pandemic. To tackle the complicated progression of the COVID-19 pandemic and to deal with the challenge of obtaining parameters reflecting reality as conditions continue to change, recent research has focused on utilizing extra information to enhance the prediction model.

One way to obtain such information is to monitor social media: Twitter, Facebook, Reddit, etc. Social networking services, which were initially simply playgrounds for small communities of computer users, have evolved into large social media platforms connecting both online and offline social networks. Several epidemic-related behaviors can be observed on social media, for instance, health information seeking, even to a heavy reliance on social media which has been observed during the COVID-19 pandemic (1, 2, 13). Several studies on the formation of pandemic waves have revealed an association between non-pharmaceutical interventions and social behaviors (14–16). With the benefit of Twitter being one of the largest social media platforms and its public posting practice, tremendous Twitter data can be utilized for big data analysis, which is attractive for COVID-19 related researches including works on predicting of COVID-19 epidemic progression, for example, using tweet counts (with relevant keywords) (17) and tweet full-text analysis (18).

Van Bavel et al. (19) observed that, especially in the current COVID-19 pandemic, “Social networks can amplify the spread of behaviors that are both harmful and beneficial during an epidemic, and these effects may spread through the network to friends, friends' friends and even friends' friends' friends.” Social networks created by popular social media platforms such as Twitter are huge and feature instant connectivity without geographical limitations. This means that popular social media platforms can amplify the spread of behaviors to a magnitude much greater than offline social networks (e.g., neighborhoods).

Several studies have revealed the emotions of social media users toward COVID-19 progression (20–24). Wheaton et al. (20) showed that “time interacting with social media did predict symptoms of depression and stress, but not anxiety or OCD symptoms.” Arora et al. (21) showed that “people with a negative sentiment are more susceptible to addictive use of social media.” Kaur et al. (24) showed in their analysis of Twitter data for February, May, and June, (2020) that the highest percentage of tweets belonged in the “Negative” category. Toriumi et al. (22) also showed in their analysis using Twitter data in Japan that social emotions toward COVID-19 from February to April, 2020 are mainly influenced by “fear”. In the work of Dyer and Kolic (23), they found “evidence of psychophysical numbing: Twitter users increasingly fixate on mortality, but in a decreasingly emotional and increasingly analytic tone.”

Furthermore, social media users are exposed to massive information with overwhelming sharing of COVID-19 related news and intentional/unintentional misinformation, which can cause severe mental health problems including high level of stress, anxiety, and contagious fear (25, 26). Moreover, regulating fake news content is still challenging (27), while COVID-19 misinformation and fake news which can exaggerate perceived risk are at highly concerned proliferation (28). Especially in Japan, the residents are at a high level of exposure to information on social media platforms, especially Twitter. In Japan, Twitter is one of the top influential social media platform with the number of monthly active users of 45 million by October 2017^1^.

Our review of previous work strongly suggests that social media platforms, including Twitter, are ideal places for monitoring, collecting, and analyzing clues that can lead to behavioral changes (29) which can help in predicting the progression of pandemics such as COVID-19. From this standpoint, we set out to design a system for predicting COVID-19 progression by utilizing Twitter data as indicators of social media reactions. We collected tweet counts related to COVID-19 as a measure of how the reactions on social media are shaped during each wave of the COVID-19 in Japan.

In addition to general tweets, we have investigated the utilization of emoji usage on Twitter to capture changes in the emotions of social media users for use in enhancing epidemiological models. Several studies have focused on capturing emotion from texts including posts on Twitter (“tweets”), for example, sentiment analysis (30) and emotion analysis (31). However, accurately understanding emotional tweets by using full-text analysis is a challenging task. Emoji analysis is an attractive approach because social media users tend to express emotions using non-verbal communication, and they share a common understanding of many emoji as several studies have shown that emojis are used on social media as non-verbal communication cues to assist communication (32–35). Emoji are digital images depicting simple illustrations including facial expressions (smiley face 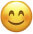, crying face 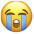, scared face 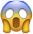, etc.). Emotional messages can be directly expressed through emoji. Because social media users share a common understanding of many emoji, emotions can be effectively and conveniently communicated through emoji. One one hand, this makes it convenient to use emoji for expressing emotional messages. One the other hand, this potentially exposes an user to a wide range of emotions with various shades of meaning, which could be overwhelming.

One crucial point when using social media data, particularly Twitter data, is that social media users may become less engaged, i.e., performing fewer actions such as “liking,” “commenting,” and “sharing,” as the pandemic lasts longer and longer (17). When engagement drops to a certain level, social media data becomes less representative of behavioral changes. The results of a study using Twitter data from the U.S. and Canada by (17) suggest that there will be less engagement through social media due to a feeling of exhaustion as waves of the pandemic continue. Therefore, in this study, we also took into consideration the results of previous studies using Japanese Twitter data.

## 2. Materials and Methods

### 2.1. Data Collection

The data consisted of tweet counts and COVID-19 infection data from Japan.

**Figure 1:**
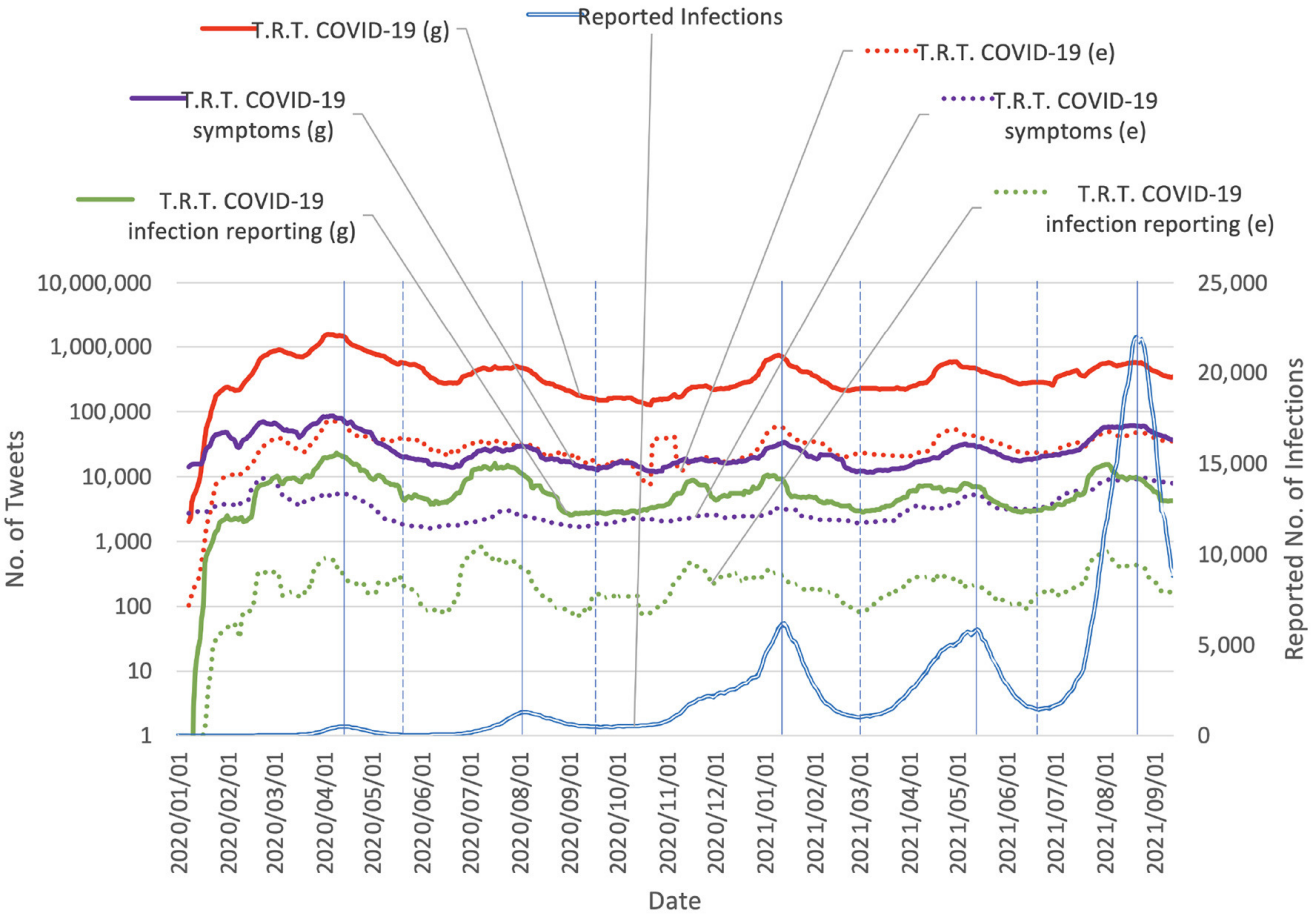
Daily chart of tweet counts vs. reported COVID-19 infections in Japan (values were smoothed by 15-day moving average). T.R.T., Tweets related to. The vertical solid lines mark the peak of the number of reported daily infections. The vertical dashed lines mark the bottom of the number of reported daily infections. The spans separated by the vertical dashed lines contain each separate wave of COVID-19. The data suggest that the number of COVID-19 related tweets has been correlated to some degree with the progression of the epidemic in Japan since the beginning of the epidemic.

The tweet count data were collected using the Twitter API (version 2) with academic research access. Several settings were considered, from the general COVID-19 related tweet count to more fine-grained target subsets of keywords. Three sets of keywords were used: COVID-19 related set, COVID-19 symptom related set^2^, and COVID-19 infection reporting related set. For each set, the collections were further filtered to retain only tweets containing emojis. The COVID-19 related set was the primary set used. The other sets were used for an ablation study and analysis of the characteristics of the tweets. The details of the settings are shown in Table 1. The collected data show that the number of COVID-19 related tweets has been correlated to some degree with the COVID-19 epidemic progression since the beginning of the epidemic (Figure 1). For analysis of tweets regarding the use of emoji, we count tweets in two categories: (g) general counting (without considering whether the tweets contain emoji or not), and (e) only count tweets containing emoji.

**Table 1:** Tweet count settings. Two categories for counting are considered: (g) general counting (of tweets whether containing emoji or not), and (e) counting of tweets containing emoji.

**Tweets Related To**	**Only Tweets with Emoji**	**Query Keywords**	**Daily No. of Tweets**
COVID-19 (g)	No	新型コロナ, コロナ感染, コロナ禍, コロナワクチン, 緊急事態宣言, まん延防止, 感染者 (*translation: [new-variant corona, corona infection, corona disaster, corona vaccine, emergency declaration, spread prevention, infected person/people]*)	414,576
COVID-19 (e)	Yes	same as above	29,484
COVID-19 symptoms (g)	No	発熱, 鼻汁, 咽頭痛, 咳嗽, 嗅覚異常, 味覚異常, 息切れ, 咳, のどの痛み, 喉の痛み, 嗅覚障害, 味覚障害,, **excluding**{風邪, インフルエンザ, 糖尿病, マラリア, サタデーナイトフィーバ, 喫煙, たばこ, アレルギー, アレルギ} (*translation: [fever, nasal discharge, sore throat, cough, dysosmia, dysgeusia, shortness of breath, cough, sore throat, sore throat, dysosmia, dysgeusia]*, *excluding: {cold, influenza, diabetes, malaria, Saturday night fever (a movie-related reference to the risk of going out dancing), smoking, tobacco, allergies, allergies }*)	28,814
COVID-19 symptoms (e)	Yes	same as above	3,597
COVID-19 infection reporting (g)	No	感染者数, 陽性者数 (*translation: [number of infected people, number of confirmed positive cases]*)	6,518
COVID-19 infection reporting (e)	Yes	same as above	232

The COVID-19 infection reporting data for Japan were obtained from JX Press^3^ The dataset contains daily infection reports for all prefectures in Japan. It was used for training or calibrating two core models used by the epidemic simulation system described in Sections 2.2 and 2.3.

### 2.2. SNS Reaction Trend and COVID-19 Epidemic Progression Change Prediction

As seen in Figure 1, throughout the waves of COVID-19, there exists a phenomenon that the reactions on Twitter also form a wave shape and each wave of the reaction on Twitter also has a correspondence to each wave of COVID-19. Given that Twitter is an influential social media platform in Japan, it is not surprising that the news about a surge in COVID-19 cases immediately results in reactions on Twitter with certain key phrases, for example, “*x* higher than last week,” and “all time high,” which quickly catches the attention of Twitter users. Based on that, we hypothesize that when the number of COVID-19 cases increases (again), the reactions on Twitter also increase. On one hand, this increases the awareness of a possible high-risk situation, which should cause people to change their behaviors and be more careful with their decisions and actions, for example, by following preventative measures including staying home, and social distancing. This may lead to a down-trend in COVID-19 infections. However, on the other hand, the massive exposure to a large amount of negative information could increase mental health problems such as experiencing excessive fear, and stress (25, 26).

**Figure 2:**
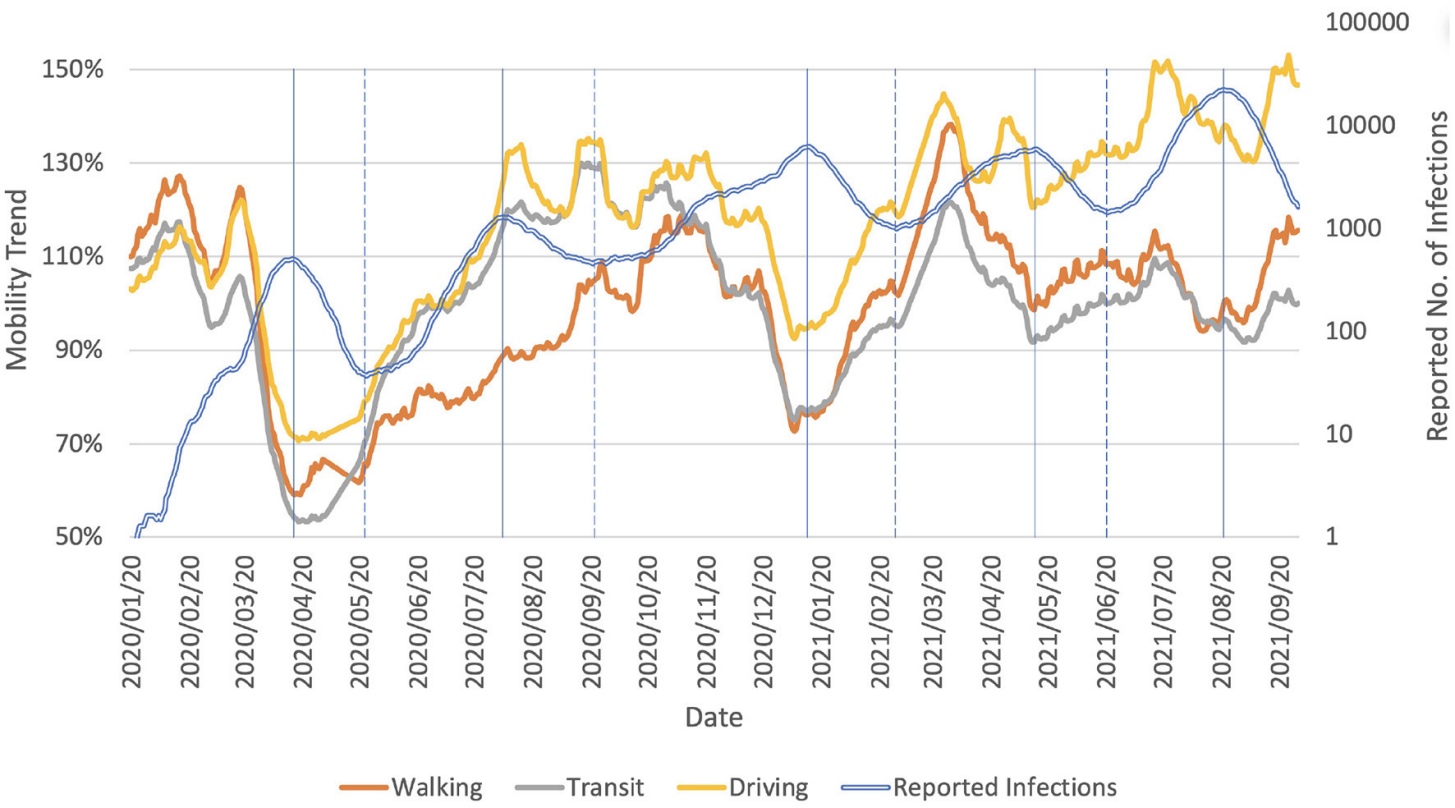
Mobility trends reports for Tokyo (23 districts), Japan. Reports are published daily and reflect requests for directions in Apple Maps. The reports show a relative volume of directions requests per country/region, sub-region, or city compared to a baseline volume on 2020/01/13. The values were smoothed by 15-day moving average. The vertical solid lines mark the peak of the number of reported daily infections. The vertical dashed lines mark the bottom of the number of reported daily infections. It is seen that in all the waves of COVID-19, the mobility is in up-trend when each COVID-19 wave is in down-trend.

A down-trend of COVID-19 infection cases could cause people to perceive a low-risk situation. As can be seen in the change of mobility, according to the mobility trends reports from Apple^4^ (Figure 2), the mobility trends up when the number of COVID-19 cases decreases, which is what happened in Japan during each of the COVID-19 waves. This indicates a tendency to relaxing some restrictions when the COVID-19 situation is perceived to be improving.

If a community remains infectious, or infectious outsiders enter into the community, the risk of another infection surge increases, and if the community perceives the situation as low-risk, another infection surge may appear, resulting in a cycle of surges and declines in the infection rate. This has been observed in the past waves of COVID-19 in Japan.

**Figure 3:**
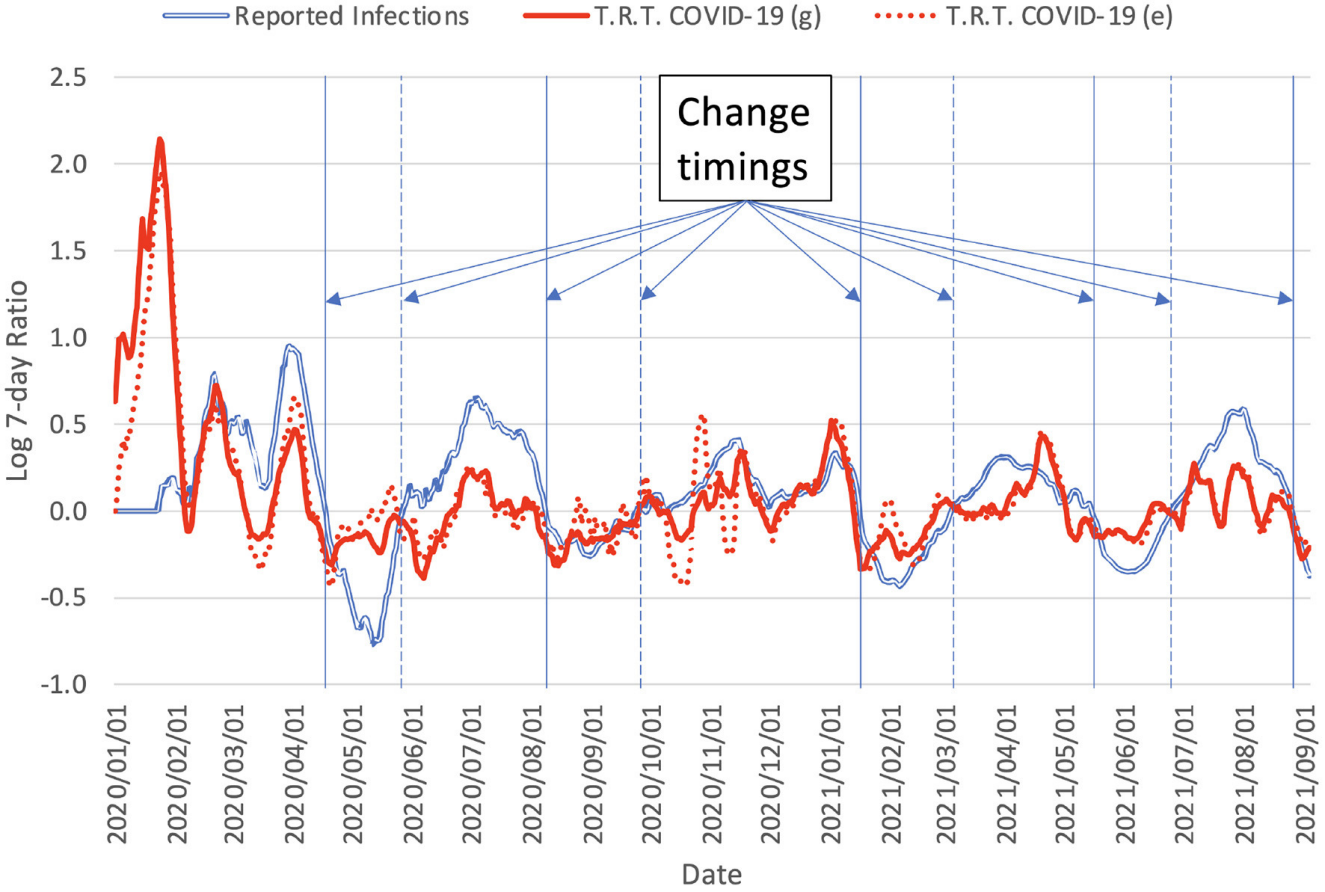
Logarithm of increasing rate of the day of the week for reported infections and tweet counts calculated using Equation (1). T.R.T., Tweets related to. The vertical solid lines mark the change of the COVID-19 trend from up-trend to down-trend (peaked out). The vertical dashed lines mark the change of the COVID-19 trend from down-trend to up-trend (infection cases start rising again). The change timings mark the moments when the logarithm of increasing rate passes the zero line: negative-to-positive indicating up-trend and positive-to-negative indicating down-trend.

As additionally shown in Figure 3, the trend in reported infections or cases was similar to the trend in the reaction level on social media. This suggests a non-negligible correlation between the two signals. Predicting the trend of changes in the epidemic progression would help to set up appropriate scenarios for simulating the future epidemic state, which in turn would support policy makers, for example, in implementing restrictions. In this sense, given the suggestion of a potential relationship between the trends of the two signals, additional information from social media reactions may further support predicting changes in the epidemic progression.

Here, the trend representations were estimated using the ratio of the signals for days *t* and *t* − 7, which were the same day of the week:


(1) $$\begin{array}{l} s_{t}=log\left(\frac{o_{t}}{o_{t-7}}\right), \end{array}$$

where *o*_*t*_ represents the two signals, the reactions on Twitter measured by tweet count and the epidemic state estimated from the reported number of new infections on day *t*, and *s*_*t*_ represents the trend measured as the 7-day change. This transformation absorbs the weekly effect observed in the Japanese data. The transformation was further smoothed by a 15-day moving average.

To model the relationship between the trend in social media reactions and the trend in epidemic progression, we utilized a long short-term memory (LSTM) neural network (36), a well-known and successful neural network architecture in time-series modeling, and the multivariate time-series of the two trends. LSTM neural networks have been used in various domains for modeling time-series and have achieved practical results. In previous studies of COVID-19 epidemic prediction systems, LSTM models were used as the core models (37–39).

To cope with the unknown complexity of the relationship between the two time-series, we use an ensemble system of multi-layer LSTM models with various hyperparameter (number of layers, number of neurons) settings and parameter initialization of the LSTM models^5^.

The LSTM system is optimized by minimizing the mean squared error:


(2) $$\begin{array}{l} MSE\left(s_{2:t},s_{2:t}^{*}\right)=\frac{1}{t-1}\overset{t}{\underset{k=2}{\sum }}\frac{1}{d}\overset{d}{\underset{j=1}{\sum }}{\left(s_{k,j}-s_{k,j}^{*}\right)}^{2}, \end{array}$$

where *t* marks the end of the observable or training data, *d* = 2 is the number of time-series (including the trend of reactions on Twitter and the trend of the epidemic progression), and s, s^*^ are the observed data and the corresponding predictions.

**Figure 4:**
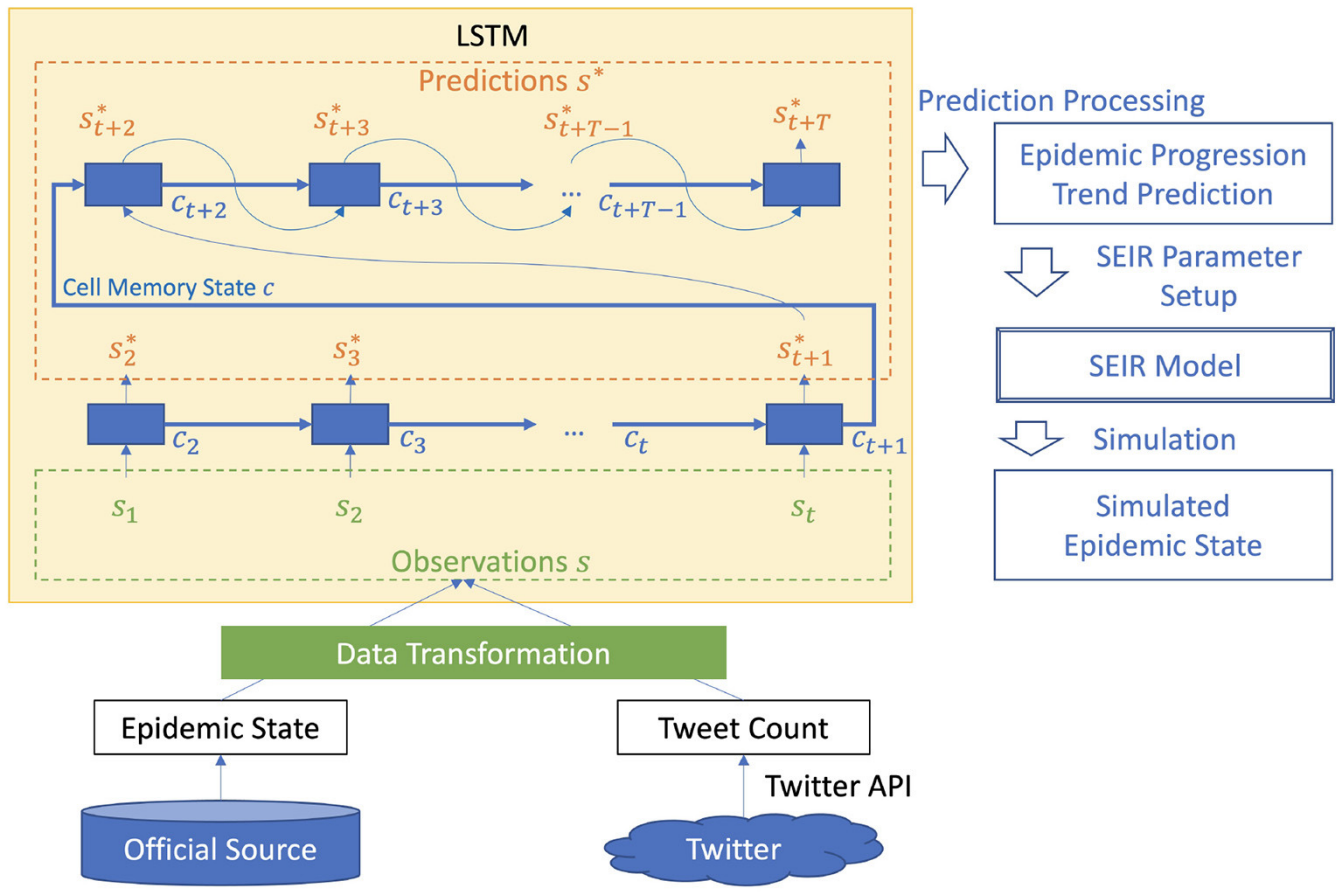
COVID-19 epidemic simulation system (*t* marks end timing of observable data).

The inference procedure has two phases. In the first phase, the LSTM ensemble system receives observed data {*s*_*k*_|*k* ∈ [1, *t*]} up to time *t* and uses them to create memory state *c*_*t*+1_ and prediction $s_{t+1}^{*}$ (Equation 3). In the second phase, from input time-step *t* + 1, the prediction of the previous time-step is used as the input to predict the next time-step (Equation 4). The inference procedure is illustrated in the “LSTM” box at the top-left of Figure 4. In the training or optimization process, only the first phase is invoked, and predictions $s_{2:t}^{*}=\left\{s_{k}^{*}|k\in \left[2,t\right]\right\}$ are used for the aforementioned optimization.


(3) $$\begin{array}{l} \left\{s_{k+1}^{*},c_{k+1}\right\}=\text{LSTM}\left(s_{k},c_{k}\right)\text{for}k\in \left[1,t\right] \end{array}$$

(4) $$\begin{array}{l} \left\{s_{k+1}^{*},c_{k+1}\right\}=\text{LSTM}\left(s_{k}^{*},c_{k}\right)\text{for}k\in \left[t+1,t+T-1\right], \end{array}$$

where *k* is the input time-step, *t* marks the end of the observable data, *T* is the length of the prediction period, *c* is the memory state of the LSTM, and s, s^*^ are the observed data and corresponding predictions.

The outputs of the *change prediction* model are used for setting up the COVID-19 simulation system described in the next subsection. The outputs of the *change prediction* model are processed to identify the timings when the predicted values change sign (illustrated in Figure 3):

- From positive to negative: the signal progression changes from increasing (up-trend) to decreasing (down-trend).
- From negative to positive: the signal progression changes from decreasing (down-trend) to increasing (up-trend).

### 2.3. COVID-19 Epidemic Simulation System

The COVID-19 epidemic simulation system consists of two stages: (1) *change prediction*, (2) *simulation*. The *change prediction* is executed as described in Section 2.3. The *simulation* is executed using SEIR, a common epidemic model. The overall flow of the system illustrated in Figure 4 is as follows.

1. *Data collection*: collect tweet count and COVID-19 epidemic state;
2. *Data transformation*: estimate trend representations for tweet count and COVID-19 epidemic progression;
3. *Change prediction*: predict trends and identify change timings;
4. *SEIR model parameter setup*: set SEIR model parameters in accordance with the identified change timings;
5. *Simulation*: perform epidemic simulation.

We used the simulation system proposed by (40) with a stochastic SEIR model to model the disease dynamics. The system supports multi-location epidemic modeling to estimate the *force of infection* (rate at which susceptible individuals are infected) by using inter-location mobility. The formulation of the SEIR model is described in the Appendix. We performed prefecture-wide multi-location setup. The SEIR model uses the following parameters: the latent period $\frac{1}{\sigma }$, which is the time interval between when an individual becomes infected and when he or she becomes infectious, the infectious period $\frac{1}{\gamma }$, which is the time interval during which an individual is infectious, and the effective reproduction number *R*_*i*_(t) for each location *i* at time *t*, which is the number of cases generated in the current state of a population.

While the latent period $\frac{1}{\sigma }$ and infectious period $\frac{1}{\gamma }$ depend on the COVID-19 variant, the effective reproduction number *R*_*i*_(*t*) depends not only on the variant but also on the contact rate in the community, which changes as the behaviors of the community members change. During one wave of the COVID-19 epidemic, the change in *R*_*i*_(*t*) was greatly affected by behavioral changes due to perceived events, e.g., surging of cases and policy changes (emergency declarations), resulting in up trends and down trends in the epidemic progression. Hence, determining *R*_*i*_(*t*) is the key to effective simulation.

A set **R**_*i*_ = {*R*_*i*_(*t*)} was obtained using the calibration method used by (40) for the period from 2020/12/24 to 2021/01/21 (the 3*rd* wave in Japan) using the observed epidemic data. Two subsets of *R*_*i*_(*t*) were established: up-trend set ${\text{R}}_{i}^{u}$ (2020/12/24–2020/01/06) and down-trend set ${\text{R}}_{i}^{d}$ (2021/01/07 – 2021/01/21).

In the *simulation* period from 2021/04/23 to 2021/06/30, for each trend (up or down) time span [*t*_*s*_, *t*_*e*_], a set of {*R*_*i*_(*t*)} for each location *i* was drawn from a uniform distribution:


(5) $$\begin{array}{l} R_{i}{\left(t\right)}_{|t_{s}\leq t\leq t_{e}}\sim U\left[m_{i}^{\left(p\right)},M_{i}^{\left(p\right)}\right], \end{array}$$

where $m_{i}^{\left(p\right)},M_{i}^{\left(p\right)}$ are, respectively, the minimum and maximum values of a set of previously obtained reproduction numbers, which can be either ${\text{R}}_{i}^{u}$ or ${\text{R}}_{i}^{d}$ depending on whether time span *p* is trending up or down. If [*t*_*s*_, *t*_*e*_] is an up-trend time span, ${\text{R}}_{i}^{u}$ is selected, and if [*t*_*s*_, *t*_*e*_] is a down-trend time span, ${\text{R}}_{i}^{d}$ is selected. The change timings, *t*_*s*_ and *t*_*e*_, are determined in the *change prediction* stage, as described in Section 2.2.

For evaluation, we measure the errors in the *change prediction* and *simulation* stages against the observed data for the period from 2021/04/23 (in the up-trend of the 4th wave) to 2021/06/30 (ending of the 4th wave). We used data from 2020/12/24 to 2021/01/21 (in the 3*rd* wave) to obtain the SEIR model parameters and data from 2020/11/15 to 2021/04/22 (the end timing of observable data) for training the *change prediction* model. Two observed timings of trend changes were used for evaluation: *t*_*a*_ = 2021/05/15 and *t*_*b*_ = 2021/06/25, where *t*_*a*_ marks the change from up-trend to down-trend, and *t*_*b*_ marks the change from down-trend to up-trend in the epidemic progression as observed in the infection reports.

The evaluation metric for *change prediction* was the difference in days Δ*days*[*t*] between the predicted date *t*′ and the actual date *t* of the trend change (Equation 6).


(6) $$\begin{array}{l} \Delta days\left[t\right]=t^{\prime }-t \end{array}$$

The evaluation metric for *simulation* was the root-mean-square error (RMSE).

## 3. Results

Table 2 shows the results for *change prediction* and *simulation*. Two baselines were used for reference.

- *Baseline 1*: *R*_*i*_(*t*) was set for the entire simulation period using **R**_*i*_ in the up-trend and down-trend periods of the 3*rd* wave. *R*_*i*_(*t*) were sampled for both the up-trend and down-trend periods without knowing the exact timing of the trend change.
- *Baseline 2*: *R*_*i*_(*t*) was set for the entire simulation period using ${\text{R}}_{i}^{u}$ in the up-trend period of the 3*rd* wave. *R*_*i*_(*t*) were sampled for only the up-trend period.

**Table 2:** Evaluation results for *change prediction* (Equation 6) and *simulation* (RMSE) for 4th wave in Japan (2021/04/23–2021/06/30) with two epidemic progression trend changes: *t*_*a*_ = 2021/05/15 and *t*_*b*_ = 2021/06/25.

**Epidemic simulation system**	**Change prediction** **(Δ*days*[*t*_*a*_]/Δ*days*[*t*_*b*_])**	**Simulation (RMSE)**	**Daily no. of tweets**
Baseline 1	n/a	18,093.9	n/a
Baseline 2	n/a	25,216.0	n/a
+*change prediction* w/o using tweet data	−16.3/−28.0	2,377.9	n/a
+*change prediction* using T.R.T. COVID-19 (g)	−7.8/−21.7	1,360.4	414,576
+*change prediction* using T.R.T. COVID-19 (e)	−8.0/−19.3	1,435.1	29,484

*Data from 2020/12/24 to 2021/01/21 were used to obtain SEIR model parameters for up-trend and down-trend periods of COVID-19 epidemic progression. Data from 2020/11/15 to 2021/04/22 were used for training change prediction model. T.R.T., Tweets related to*.

For our approach, we used three system settings:

- +*change prediction* w/o using tweet data: the epidemic simulation system was setup with*change prediction* using only the epidemic state data, not the tweet data.
- +*change prediction* using T.R.T. COVID-19 (g): the epidemic simulation system was setup with *change prediction* using both the epidemic state data and the COVID-19 related tweet count data.
- +*change prediction* using T.R.T. COVID-19 (e): similar to setting for (g) except that tweets were filtered to remove ones not containing emoji.

The additional use of the COVID-19 related tweet count (g) resulted in better prediction of the epidemic progression trend changes than without using the count: prediction was improved by 8.5 days for *t*_*a*_ and 6.3 days for *t*_*b*_. This led to a reduction of 42.8% in the RMSE. Given that the daily tweet count of COVID-19 related tweets filtered for emoji (e) was 92.9% smaller than the more general count (g), the results are similar: the difference in *change prediction* was 0.2 days for *t*_*a*_ and 2.4 days for *t*_*b*_, and the RMSE was 5.5% worse. In all results, the predicted trend changes preceded the observed changes. The baseline results show that without estimating the trending change, the RMSE were 7.6–18.5 times worse.

## 4. Discussion

The relationship between user reactions on social media and the COVID-19 epidemic progression remains close for the long term. Social media engagements related to COVID-19 have remained fairly steady over the five waves of COVID-19 epidemic surges in Japan. They reached their highest level in the first wave, dropped a bit in the second wave, and then picked up in the following waves. The engagements peaked at around the peak of each wave. This demonstrates the value of using epidemic-related social media data, particularly Twitter data.

The 3rd and 4th waves in the period from 2020/11/15 to 2021/06/25 exhibited similar characteristics: the wave shapes were similar (Figure 1) and the vaccination rates were similar^6^. Despite the similar wave shapes, the reactions to non-pharmaceutical interventions and emergency declarations differed between the two waves. In the 3*rd* wave, an emergency declaration was issued on 2021/01/07, and a change in the epidemic progression trend (from increasing to decreasing) was observed on 2021/01/17 (10 days later). In contrast, in the 4th wave, an emergency declaration was issued on 2021/04/25, and a change in the epidemic progression trend was observed on 2021/05/15 (20 days later). The 10-day later response in the 4th wave may be attributed to reluctance to comply or exhaustion after already being subjected to two previous emergency declarations which imposed a great level of stress and anxiety (41, 42). The reluctance or exhaustion level can be somewhat correlated with the reactions on social media when users choose to share their emotional thoughts to others, which provides informative features to our *change prediction* model and resulted in more accurate prediction of the change in the epidemic progression trend compared with the setting of not using social media data.

As demonstrated in the results (Table 2), the ability to predict the change timings including both the down-trend and up-trend timings for the 4th waves shows that the *change prediction* model learns to indicate that there exists the repetitive phenomenon in the reactions on Twitter and the COVID-19 progression. With the prediction, the model indicates that the next progression will also come in a wave shape. The repetitive phenomenon, however, could disappear or become undetectable if the community is no longer infectious, or no more infectious outsiders enter the community or there is no more reporting of the epidemic situation. Reaching the peak of a wave early or late mainly depends on community members' perception of the epidemic situation. As one major information sharing channel, social media including Twitter plays an important role in amplifying the impact of information availability which directly affects the perception of the epidemic situation. As this continues, the model can be useful for predicting the appearance of the phenomenon in the form of change of reaction trends on social media and COVID-19 progression trends.

From the results, we can see the challenges in predicting the exact timings of these events of the trend changes. The accuracy reduces as the time is further in the future. the first timing is predicted with 7.8–8.0 days difference, but the next timing is predicted with 19.3–21.7 days difference from the observed timings. All predictions show earlier timings than the observed ones. The challenges can be attributed to the change of COVID-19 variants or the change of society's perception of COVID-19 situation. This could be considered by deeply analyzing the tweets in term of their contents and their networks of tens to hundreds of millions of tweets or even more if possible relevant aspects other than COVID-19 are necessary to collect.

###  The 6th Wave of COVID-19 in Japan

**Figure 5:**
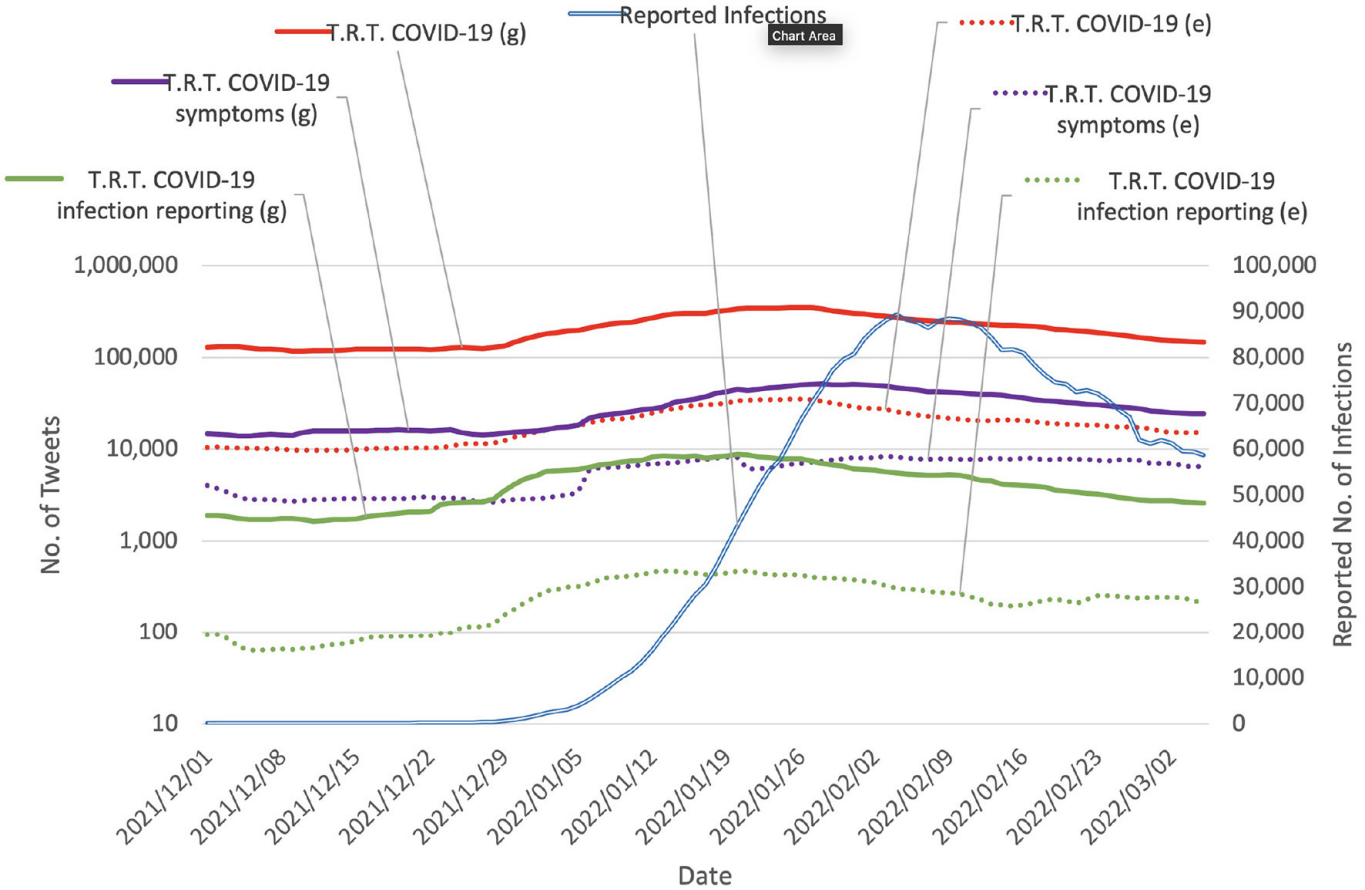
Daily chart of tweet counts vs. reported COVID-19 infections in the 6th wave of COVID-19 in Japan (values were smoothed by 15-day moving average). T.R.T., Tweets related to.

Since the end of 2021 and the start of 2022, Japan has been facing the 6th wave of COVID-19 with the emerging of the Omicron variant^7^. It once again triggers another wave of reactions on Twitter (Figure 5). To illustrate the applicability of our method to this new situation, we evaluate the prediction of the change timing of the COVID-19 progression trend from up-trend to down-trend as actually observed on $t_{a}^{\left(6\right)}=2022/02/10$. The results (Table 3) show that the additional use of the COVID-19 related tweet count (g) resulted in better prediction of the epidemic progression trend changes than without using the count: prediction was improved by 5.7 days. This led to a reduction of 37.4% in the RMSE of COVID-19 case simulation. The evaluation of the method is relatively similar in both the 4th and 6th waves. This suggests that the social media reactions still remain in an effective relationship with the COVID-19 progression in the recent situation.

**Table 3:** Evaluation results for *change prediction* (Equation 6) and *simulation* (RMSE) in the 6th wave in Japan (2022/01/01–2022/03/05) with the epidemic progression trend change observed on $t_{a}^{\left(6\right)}=2022/02/10$.

**Epidemic simulation system**	**Change prediction** **($\Delta days\left[t_{a}^{\left(6\right)}\right]$)**	**Simulation (RMSE)**
Baseline 1	n/a	322,075.6
Baseline 2	n/a	523,815.0
+*change prediction* w/o using tweet data	−17.1	53,849.5
+*change prediction* using T.R.T. COVID-19 (g)	−11.4	33,732.1
+*change prediction* using T.R.T. COVID-19 (e)	−11.9	33,864.9

*Data from 2020/12/24 to 2021/01/21 were used to obtain SEIR model parameters for up-trend and down-trend periods of COVID-19 epidemic progression with an adjustment of the basic reproduction number for the infectious power of the Omicron variant using observed data from 2022/01/01 to 2022/01/14. Simulation RMSE was evaluated during the period from 2022/01/15 to 2022/03/05. Data from 2020/11/15 to 2021/12/22 were used for training change prediction model. (T.R.T., Tweets related to)*.

###  Future Direction

For further improvement in the *simulation* results, the method for setting the SEIR model parameters needs to be further improved, especially for the setting of *R*_*i*_(*t*). In this study, the distribution from which the set of {*R*_*i*_(*t*)} for each location *i* was drawn was assumed to be uniform, and the up- and down-trend parameter sets were manually established. The setting of the SEIR model parameters would be more challenging in periods in which the epidemic conditions greatly differed, e.g., the 5th and 6th waves in Japan with the dominance of the Delta and Omicron variants, respectively. Viable options include selecting values from the most recent wave with adjustment for the infectious power of newer variants and selecting from the period with the most similar social media reactions although measuring similarity would be a challenging task. Furthermore, it is necessary to consider the emergence of new COVID-19 variants and how they would affect the parameters as well as the social media reactions. These challenges will be addressed in future work.

As preparation for future work, we performed experiments on training the *change prediction* model using different fine-grained tweet counts:

- T.R.T. COVID-19 symptoms(g),
- T.R.T. COVID-19 symptoms(e),
- T.R.T. COVID-19 infection reporting (g),
- T.R.T. COVID-19 infection reporting (e).

The tweet counts are listed in Table 1, and the results of the additional experiments are shown in Table 4.

**Table 4:** Tweet counts for *change prediction* for 4th wave in Japan (2021/04/23–2021/06/30) with two epidemic progression trend changes: *t*_*a*_ = 2021/05/15 and *t*_*b*_ = 2021/06/25.

**Tweet count for *change prediction***	**Change prediction** **(Δ*days*[*t*_*a*_] / Δ*days*[*t*_*b*_])**	**Simulation (RMSE)**	**Daily no. of tweets**
T.R.T. COVID-19 (g)	−7.8/−21.7	1,360.4	414,576
T.R.T. COVID-19 (e)	−8.0/−19.3	1,435.1	29,484
T.R.T. COVID-19 symptoms (g)	−17.4/−27.7	2,478.9	28,814
T.R.T. COVID-19 symptoms (e)	−16.2/−29.4	2,389.6	3,597
T.R.T. COVID-19 infection reporting (g)	−13.4/−26.4	2,051.2	6,518
T.R.T. COVID-19 infection reporting (e)	−12.4/−23.4	1,932.7	232

*Data from 2020/12/24 to 2021/01/21 were used to obtain SEIR model parameters for up-trend and down-trend periods of COVID-19 epidemic progression. Data from 2020/11/15 to 2021/04/22 were used for training change prediction model. (T.R.T., Tweets related to)*.

Compared with using the general-topic COVID-19 related tweet counts, using more specific-topic tweet counts did not show improvement: the RMSE was 34.7–82.2% worse for the *simulation* period. This suggests that the relationship between reactions on social media and epidemic progression is complex. The general count, covering a broad range of topics, exhibited greater predictive power than the more specific counts. Manual topic design thus may not be an efficient approach. The development of automatic topic discovery techniques for finding relevant topics discussed on social media that can support epidemic progression prediction could be promising.

The results for tweet counts with emoji filtering (e) compared with the general tweet counts (g) showed that the emoji settings have similar representative value as the general settings: the RMSE difference was only 3.6–5.8% even with 87.5–96.4% fewer tweets. One advantage of using emoji settings is the ability to perform fine-grained analysis on specific emotions (fear, anger, etc.) represented by various emojis. Further studies on the specific emotions used by social media users for typical topics could help in discovering topics where changes in emotion could affect epidemic progression. This could be done by analyzing social media contents (emoji vs. topics) to identify emotions trending on topics relevant to epidemic progression. This is left for future work.

This work contributes its results to the demonstration of the necessity of big social media data analysis in crucial worldwide problems including dealing with pandemics. Together with medical big data and wearable Internet of Medical Things (43–45) which have the ability to monitor the physical conditions of patients, big social media data analysis can help with detecting mental health problems in the society. On one hand, real-time COVID-19 symptom data with smart data fusion can be gathered instantaneously by using wearable sensors potentially artificial intelligence-enabled placed on the patient's body. They could be powered with advanced deep learning and cloud computing for quick, early, and efficient treatment for individuals, thus in turn improving public health care. On the other hand, the similar technology of deep learning and cloud computing can also be utilized for processing big social media data including user interactions to not only detect the individual mental health problems but can also detect the change of social mental states.

## 5. Conclusion

We have presented an approach to predicting COVID-19 epidemic progression that utilizes data from Twitter, one of the most influential social media platforms worldwide. We demonstrated the effectiveness of this approach in a case study for Japan where Twitter is one of the most influential social media platforms. Preliminary revealed that the reaction trends on Twitter showed a repetitive phenomenon over all the waves of COVID-19 in Japan: the trends in social reactions matched those in the COVID-19 epidemic progression for the majority of the time. From that observation, we designed a system that utilizes neural networks for time-series modeling and exploits the reactions represented by tweet counts to predict changes in the trend of COVID-19 epidemic progression. Our experimental results show that it is possible to predict the trends in COVID-19 infections from the trends in the reactions on Twitter. This means that it is important to pay attention to the evolution of mass social media platforms and their effects on critical events including pandemics. However, it may be challenging to identify crucial factors from Twitter data that can be decisive clues to changes in the COVID-19 progression trend. We will address this problem by not simply focusing on the tweet count but rather by analyzing the massive amounts of Twitter data (tens to hundreds of millions of tweets), including the tweet contents and the network of tweets.

## Data Availability Statement

The data analyzed in this study is subject to the following licenses/restrictions: Twitter's Developer Agreement and Policy, JX Press' License for Research Purposes, and ZENRIN DataCom's License for Research Purposes. Requests to access these datasets should be directed to https://twitter.com/, https://jxpress.net/, and https://www.zenrin-datacom.net/.

## Author Contributions

VT and TM contributed to the conception and design of the study and to the data collection. VT implemented the system, performed data curation, conducted the experiments, and wrote the first draft of the manuscript. TM validated the progress and results of the study *via* daily discussion with VT. Both authors contributed to manuscript revision and read and approved the submitted version.

## Funding

This work was supported with funding from the COVID-19 Program and the Future Investment Program of the Research Organization of Information and Systems, Japan.

## Conflict of Interest

The authors declare that the research was conducted in the absence of any commercial or financial relationships that could be construed as a potential conflict of interest.

## Publisher's Note

All claims expressed in this article are solely those of the authors and do not necessarily represent those of their affiliated organizations, or those of the publisher, the editors and the reviewers. Any product that may be evaluated in this article, or claim that may be made by its manufacturer, is not guaranteed or endorsed by the publisher.

## Footnotes

^1^ https://twitter.com/TwitterJP/status/923671036758958080

^2^The symptom-related keywords were obtained from https://www.kansensho.or.jp/ref/d77.html and (17).

^3^ https://jxpress.net/

^4^ https://covid19.apple.com/mobility

^5^no. of layers ∈ {1, 2, 3, 4} × no. of neurons ∈ {4, 8, 16} × no. of initializations ∈ {128}.

^6^ https://www.kantei.go.jp/jp/headline/kansensho/vaccine.html

^7^ https://www.niid.go.jp/niid/en/2019-ncov-e.html

## Appendix

In this study, we used the simulation system proposed by (40) with a stochastic SEIR model used to model the disease dynamics. This system supports multi-location epidemic modeling to estimate the *force of infection* using inter-location mobility. For Japan, we performed prefecture-wide multi-location setup. Given the parameters, including the reproduction numbers *R*_*i*_(t), latent period $\frac{1}{\sigma }$, and infectious period $\frac{1}{\gamma }$, the transitions between the compartments **S**usceptible, **E**xposed, **I**nfected, and **R**ecovered for each location *i* are

(7)
$$\begin{array}{l} N_{S_{i}\to E_{i}}\left(t\right)=\\ \text{Binom}\left(S_{i},1-\text{exp}\left(-\Delta t\cdot {\text{FOI}}_{i}\left(t\right)\right)\right) \end{array}$$

(8)
$$\begin{array}{l} N_{E_{i}\to I_{i}^{\left(1\right)}}\left(t\right)=\text{Binom}\left(E_{i},1-\text{exp}\left(-\Delta t\cdot \sigma \right)\right) \end{array}$$

(9)
$$\begin{array}{l} N_{I_{i}^{\left(1\right)}\to I_{i}^{\left(2\right)}}\left(t\right)=\text{Binom}\left(I_{i}^{\left(1\right)},1-\text{exp}\left(-\Delta t\cdot \gamma^{\prime }\right)\right) \end{array}$$

(10)
$$\begin{array}{l} N_{I_{i}^{\left(2\right)}\to I_{i}^{\left(3\right)}}\left(t\right)=\text{Binom}\left(I_{i}^{\left(2\right)},1-\text{exp}\left(-\Delta t\cdot \gamma^{\prime }\right)\right) \end{array}$$

(11)
$$\begin{array}{l} N_{I_{i}^{\left(3\right)}\to R_{i}}\left(t\right)=\text{Binom}\left(I_{i}^{\left(3\right)},1-\text{exp}\left(-\Delta t\cdot \gamma^{\prime }\right)\right) \end{array}$$

(12)
$$\begin{array}{l} \gamma^{\prime }=\gamma \cdot k \end{array}$$

(13)
$$\begin{array}{ll} {\text{FOI}}_{i}\left(t\right)=\left(1-\underset{j\neq i}{\sum }p_{a}\frac{M_{i,j}}{H_{i}}\right) & \cdot {\text{FOI'}}_{i}\left(t\right)+\underset{j\neq i}{\sum }\left(p_{a}\frac{M_{i,j}}{H_{i}}\cdot {\text{FOI'}}_{j}\left(t\right)\right) \end{array}$$

(14)
$$\begin{array}{l} {\text{FOI'}}_{i}\left(t\right)=\beta_{i}\left(t\right)\frac{I_{i}{\left(t\right)}^{\alpha }}{H_{i}} \end{array}$$

(15)
$$\begin{array}{l} \beta_{i}\left(t\right)=R_{i}\left(t\right)\cdot \gamma \end{array}$$

(16)
$$\begin{array}{l} I_{i}\left(t\right)=\overset{k=3}{\underset{j=1}{\sum }}I_{i}^{\left(j\right)}\left(t\right), \end{array}$$

where *M*_*i,j*_ represent the daily mobility from location *i* to location *j*, *H*_*i*_ is the population of location *i*, *p*_*a*_ is the proportion of time that moving individuals spend away, and α is the mixing coefficient.
